# Trends and associated factors of mental health diagnoses in Catalan Primary Care (2010–2019)

**DOI:** 10.1192/j.eurpsy.2024.1793

**Published:** 2024-12-10

**Authors:** Ariadna Mas, Derek Clougher, Gerard Anmella, Clàudia Valenzuela-Pascual, Michele De Prisco, Vincenzo Oliva, Giovanna Fico, Iria Grande, Ivette Morilla, Xavier Segú, Mireia Primé-Tous, Victoria Ruíz, María Antonieta Also, Sandra Murgui, Elisenda Sant, Mireia Sans-Corrales, Miquel Àngel Fullana, Antoni Sisó-Almirall, Joaquim Radua, Jordi Blanch, Myriam Cavero, Eduard Vieta, Diego Hidalgo-Mazzei

**Affiliations:** 1 Department of Psychiatry and Psychology, Hospital Clínic de Barcelona, Catalonia, Barcelona, Spain; 2 Bipolar and Depressive Disorders Unit, Institut d’Investigacions Biomèdiques August Pi I Sunyer (IDIBAPS), Catalonia, Barcelona, Spain; 3 Biomedical Research Networking Centre Consortium on Mental Health (CIBERSAM), Instituto de Salud Carlos III, Madrid, Spain; 4 Department of Medicine, School of Medicine and Health Sciences, Institute of Neurosciences (UBNeuro), University of Barcelona (UB), Catalonia, Barcelona, Spain; 5 BIOARABA, Department of Psychiatry. Hospital Universitario de Alava. CIBERSAM. University of the Basque Country, Vitoria, Spain; 6 Consorci d’Atenció Primaria de Salut Barcelona Esquerra (CAPSBE), Barcelona, Spain; 7 Centre for Affective Disorders (CfAD), Institute of Psychiatry, Psychology and Neuroscience (IoPPN), King’s College London, London, UK

**Keywords:** Catalonia, mental health diagnoses, prevalence, primary care, stress

## Abstract

**Background:**

The prevalence of mental health disorders has significantly increased in recent years, posing substantial challenges to healthcare systems worldwide, particularly primary care (PC) settings. This study examines trends in mental health diagnoses in PC settings in Catalonia from 2010 to 2019 and identifies associated sociodemographic, clinical characteristics, psychopharmacological treatments, and resource utilization patterns.

**Methods:**

Data from 947,698 individuals without prior severe mental illness, derived from the Data Analytics Program for Health Research and Innovation (PADRIS), were analyzed for this study. Sociodemographic data, diagnoses, and resource utilization were extracted from electronic health records. Descriptive statistics, chi-square tests, Mann–Whitney tests, and a multivariate binary logistic regression were employed to analyze the data.

**Results:**

Over the study period, 172,112 individuals (18.2%) received at least one mental health diagnosis in PC, with unspecified anxiety disorder (40.5%), insomnia (15.7%) and unspecified depressive disorder (10.2%) being the most prevalent. The prevalence of these diagnoses increased steadily until 2015 and stabilized thereafter. Significant associations were found between mental health diagnoses, female sex, lower socioeconomic status, higher BMI, and smoking status in a multivariate binary logistic regression.

**Conclusions:**

This study highlights a growing burden of stress-related mental health diagnoses in PC in Catalonia, driven by demographic and socioeconomic factors. These findings may be indicative of broader trends across Europe and globally. Addressing this rising prevalence requires innovative approaches and collaborative strategies that extend beyond traditional healthcare resources. Engaging stakeholders is essential for implementing effective, sustainable solutions that promote mental health in Catalonia and potentially inform similar initiatives worldwide.

## Introduction

Over the past two decades, a substantial body of empirical research has documented a marked increase in the prevalence and societal burden of mental health disorders, particularly anxiety and depressive related conditions. This escalation has placed considerable strain on the extant health care resources, which were already operating at capacity, both within the European context and on a global scale [1,2]. Environmental stressors such as economic crises have exacerbated this already challenging context [3–5].

Beyond these temporary crises, the rise in mental health issues has been attributed to factors such as population growth, climate change, immigration, socioeconomic disparities, urbanization all of which contribute to increasing general levels of psychological stress [6,7]. Concurrently, psychological stress is increasingly identified by the ample availability of screening tools, symptom checklists, and greater public awareness, which can lead to the overuse of terms like ‘anxiety’ and ‘depression’ in contexts that do not necessarily reflect clinical disorders [8]. Further, the perception of sub-threshold psychological stress as a mental health disorder is believed to have contributed to its increasing medicalization by healthcare providers [9].

Primary care (PC) is the first line in coping with this growing mental health demand, where we have previously reported a steady increase in antidepressant (AD) prescriptions. While this increase may not be fully explained by a corresponding rise in recorded mental health diagnoses warranting AD use, it’s important to note that changes in diagnostic practices, increased awareness, and reduced stigma could all contribute to evolving patterns of both diagnoses and prescriptions [10]. Numerous studies have highlighted that mental health care continuously and significantly strains the limited resources of PC settings, both in Spain and globally [11,12]. In Spain, this burden has been primarily attributed to the high prevalence rates of anxiety and depressive diagnoses in PC [13,14].

To our knowledge, no recent data, including the evolution of mental health diagnoses prevalence in PC, its determinants and associated resource use in PC from the last decade have been published. Hence, this study aims to (1) describe trends in mental health diagnoses in PC from 2010 to 2019 in Catalonia, and (2) characterize and compare differences among individuals who did and did not receive a mental health diagnosis based on sociodemographic, clinical characteristics, psychopharmacological treatments, and resource utilization. Additionally, we aim to determine the most relevant factors contributing to the likelihood of receiving a mental health diagnosis in PC during the study period.

## Methods

For this study, data was procured from the Data Analytics Program for Health Research and Innovation (PADRIS) of the Agency for Health Quality and Assessment of Catalonia (AQuAS). This data, which was anonymized and de-identified, was obtained in strict adherence to the prevailing legal and regulatory framework, ethical guidelines, and principles of transparency. PADRIS routinely accesses comprehensive individual-level data encompassing demographic and socioeconomic characteristics, as well as extensive health-related and service-use data, which is generated from patient consultations within the public Catalan healthcare system (CatSalut) [15]. CatSalut provides universal public health coverage to all residents of Catalonia, Spain.

The database included 947,698 subjects who sought PC between 2010 and 2019 without any contact with public mental health services during this period. This cohort, comprising both children and adults without age restrictions, constitutes the control group of the PRESTO project (www.prestoclinic.cat) [16]. It was matched by age, sex, and health region with 479,000 individuals who consulted specialized mental health services of CatSalut. Individuals were excluded if they received a mental health diagnosis or accessed any specialized mental health care service (outpatient, inpatient, or emergency room) at any point during the study period. Sociodemographic information, diagnoses, resource use, and cumulative prescriptions of psychotropic drugs for these individuals were retrospectively gathered from electronic health records (EHR), with sociodemographic and clinical variables captured as of December 2019. The data covered the period from January 2010 to December 2019, with diagnoses and resource use tracked throughout this timeframe, while sociodemographic and clinical information reflected the status at the study’s endpoint.

Diagnoses considered in this study were coded according to the International Statistical Classification of Diseases and Related Health Problems 10th Revision (ICD-10). Previous diagnoses recorded under the ICD-9 classification system were recoded to the ICD-10 classification to ensure consistency and accuracy in disease classification throughout the study period with the eCIEMaps v4.0.05 tool provided by the Spanish Health Ministry (https://www.eciemaps.sanidad.gob.es/mapping) [17].

For the purposes of this specific investigation, only data from the control sample who did not have a previously registered consultation at the public specialized mental health services were considered in the analyses. Our primary outcome was the presence of a mental health diagnosis made by the PC during the study period. When multiple diagnoses throughout the study period were registered, mood and anxiety disorders were prioritized as a primary diagnosis, when not, the most recent diagnosis was considered. Only one diagnosis per subject was accounted for the frequency analyses. We considered any mental disorder except for neurodevelopmental disorders, neurocognitive disorders, and nicotine dependence among the disorders due to substance use or addictive behaviours eligible for inclusion. This decision was made to exclude mental health conditions that are more typically associated with long-term trajectories that might have preceded the study timeframe. Additionally, by excluding these conditions, we aimed to maintain a clear and specific focus on newly arising mental health diagnoses, thereby ensuring the relevance and specificity of our findings. In the case of nicotine dependence, which is often a fluctuating condition due to its nature, we utilized individuals’ smoking status as a reliable measure. This status, which categorizes individuals as a ‘Nonsmoker’, ‘Smoker’, or ‘Ex Smoker’, was consistently evaluated and recorded as a distinct variable.

To assess the distribution of continuous and count variables, we performed visual analyses (i.e., histograms and Q-Q plots), which revealed significant deviations from normality in several variables, primarily due to skewness and a high proportion of zero values. Consequently, despite our large sample size, we employed non-parametric Mann–Whitney tests for non-normal continuous variables, including BMI, number of medical comorbidities, and medication prescriptions. For categorical variables, chi-square tests were used. This approach ensures a more conservative analysis approach, particularly for variables that do not meet the assumptions of parametric tests, even in large samples. To identify the variables that might contribute to having a mental health diagnosis during the study period, we conducted a multivariate binary logistic regression considering having or not having a mental health diagnosis during the study period as the dependent variable and sex, age, nationality, socioeconomic levels, healthcare region, body mass index (BMI), smoking status and number of medical comorbidities as covariates. The level of significance was set at *p* < 0.05 for all tests. All analyses were conducted using R 4.3.1.

## Results

**Figure 1.**
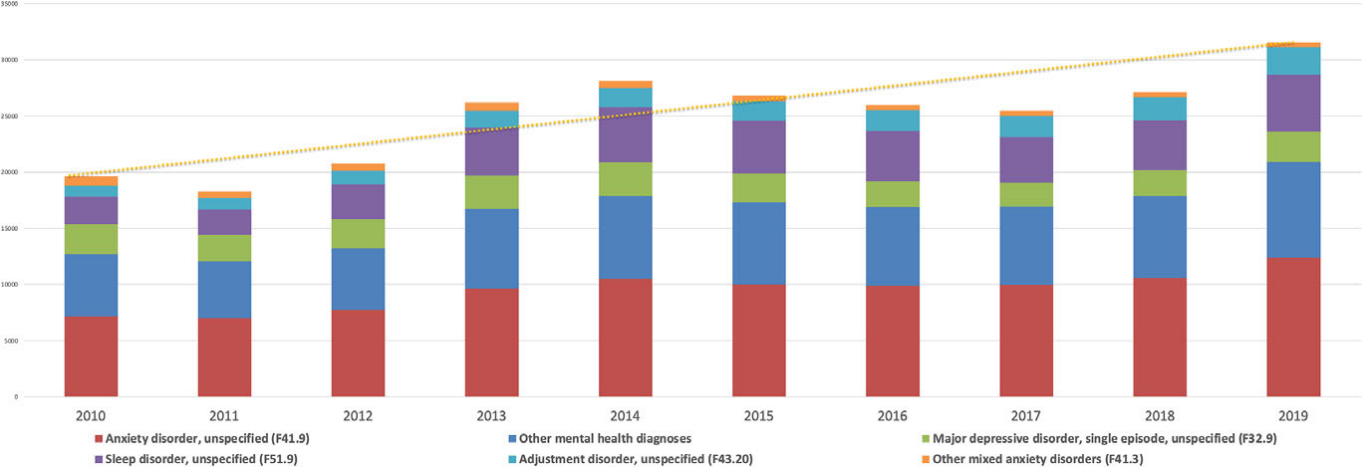
Most prevalent mental health diagnoses in primary care from 2010–2019.

A total of 947,698 subjects (50.9% females and 31.8% middle-aged adults) were included in our study (Table 1). During the study period, a total of 250,044 mental health diagnoses were recorded in PC among the considered sample. Specifically, 172,112 subjects (18.2%) received at least one mental health diagnosis with a mean of 1.4 (SD = 0.81) diagnoses per subject. The most prevalent primary diagnoses during the study period were unspecified anxiety disorder (F41.9) (40.5%), other insomnia not due to a substance or known physiological condition (F51.09) (15.7%), unspecified major depressive disorder, single episode (F32.9) (10.2%), unspecified adjustment disorder (F43.20) (6.2%) and other anxiety disorders (F41.3) (2.3%) (Table 2). Considering our exclusion criteria, 9% (i.e., neurodevelopmental disorders and nicotine dependence) of all mental health diagnoses were excluded. Over the years, there was an increase in the prevalence of these five most prevalent diagnoses in contrast to all other mental health diagnoses. This increase was particularly evident from 2011 to 2015 at an average of 10.7% per year, after which the frequency of these diagnoses maintained a plateau without any subsequent significant decline afterward (Figure 1).

**Table 1.** Sociodemographic, clinical characteristics, and resources use of the sample 2010–2019

		Any mental health diagnosis in Primary Care				***χ*** ^**2**^	*p*
		No		Yes			
		N	%	N	%		
Sex	Male	380,698	49.1%	58575	34.0%	12,832.92	<0.001
	Female	394,888	50.9%	113537	66.0%		
Age	Children (0–12)	99,490	12.8%	17874	10.4%	1,209.62	<0.001
	Adolescents (13–17)	92,565	11.9%	19495	11.3%		
	Young Adults (18–24)	61,841	8.0%	13771	8.0%		
	Early Adults (25–34)	65,790	8.5%	13667	7.9%		
	Mid-Range Adults (35–44)	100,123	12.9%	21948	12.8%		
	Middle-age Adults (45–64)	230,507	29.7%	54702	31.8%		
	Older Adults (65+)	125,270	16.2%	30655	17.8%		
Nationality	Spanish	640,780	82.6%	155550	90.4%	6,316.64	<0.001
	Non-Spanish	134,806	17.4%	16562	9.6%		
Healthcare region	Alt Pirineu	8,604	1.1%	1456	0.8%	826.38	<0.001
	Barcelona	512,259	66.0%	117558	68.3%		
	Tarragona	55,875	7.2%	9725	5.7%		
	Central Catalonia	62,232	8.0%	13873	8.1%		
	Girona	76,324	9.8%	15497	9.0%		
	Lleida	43,154	5.6%	9841	5.7%		
	Terres de l’Ebre	17,138	2.2%	4162	2.4%		
Socioeconomic status^*^	Low	389,728	50.2%	85174	49.5%	6,240.80	<0.001
	Moderate	294,495	38.0%	57119	33.2%		
	High	10,476	1.4%	1098	0.6%		
Smoking status	Non-smoker	235,535	66.4%	78578	62.5%	627.34	<0.001
	Smoker	86,515	24.4%	33695	26.8%		
	Ex-Smoker	32,793	9.2%	13413	10.7%		
		**Median**	**IQR**	**Median**	**IQR**	**U**	***p***
Body Mass Index (BMI)		26.04	8.81	27.74	7.61	6.98 x 10^8	*p* < 0.001
Medical Comorbidities		0.00	1.00	1.00	3.00	4.66 x 10^10	*p* < 0.001
Primary Care visits		25.00	41.00	50.00	54.00	3.93 x 10^10	*p* < 0.001
Benzodiazepines prescriptions (DDD)		30	130	79	386	4.68 x 10^8	*p* < 0.001
Antidepressants prescriptions (DDD)		270	1,608	364	1,372	1.69 x 10^9	*p* < 0.001
Antipsychotics prescriptions (DDD)		2	13	5	44	1.17 x 10^8	*p* < 0.001

IQR = Interquartile range, DDD = defined daily dose, U = Mann–Whitney statistic.

* Tax-exempt status is not listed in the table.

**Table 2.** Most frequent mental health diagnoses in primary care in Catalonia 2010–2019

Diagnosis	N	%
Unspecified anxiety disorder (F41.9)	69,623	40.5
Insomnia not due to known substance or physiological condition (F51.09)	26,936	15.7
Unspecified major depressive disorder, single episode (F32.9)	17,501	10.2
Unspecified adjustment disorder (F43.20)	10,609	6.2
Other mixed anxiety disorders (F41.3)	4,028	2.3
Generalized anxiety disorder (F41.1)	3,213	1.9
Behavioral disorder, unspecified (F98.9)	3,164	1.8
Alcohol abuse, uncomplicated (F10.10)	2,351	1.4
Other somatoform disorders (F45.8)	2,278	1.3
Dysthymic disorder (F34.1)	1,900	1.1
Alcohol dependence, uncomplicated (F10.20)	1,667	1.0
Other specified phobias (F40.8)	1,438	0.8
Eating disorder, unspecified (F50.9)	928	0.5
Alcohol use, unspecified (F10.9)	841	0.5
Major depressive disorder, single episode, mild (F32.0)	840	0.5
Phobic anxiety disorder, unspecified (F40.9)	720	0.4
Major depressive disorder, single episode, severe without psychotic symptoms (F32.2)	711	0.4
Panic disorder without agoraphobia (F41.0)	656	0.4
Cannabis abuse, uncomplicated (F12.10)	612	0.4
Acute stress reaction (F43.0)	599	0.3
Other specified behavioral and emotional disorders with onset usually occurring in childhood and adolescence (F98.8)	539	0.3
Other mental health disorders	20,958	12.1

Significant statistical differences in sociodemographic characteristics were identified when comparing people who received at least one mental health diagnosis in PC to those who did not during the study timeframe. Specifically, variables associated with having a mental health diagnosis included older age (*χ* ^2^ = 1,209.62, *df* = 6, *p* < 0.001, Cramér’s *V* = 0.036), female sex (*χ* ^2^ = 12,832.92, *df* = 1, *p* < 0.001, Cramér’s *V* = 0.116), Spanish nationality (*χ* ^2^ = 6,316.64, *df* = 1, *p* < 0.001, Cramér’s *V* = 0.082), lower socioeconomic status (*χ* ^2^ = 6,240.80, *df* = 3, *p* < 0.001, Cramér’s *V* = 0.081), smoker or ex-smoker (*χ* ^2^ = 14,004.50, *df* = 2, *p* < 0.001, Cramér’s *V* = 0.122), and predominantly from urban regions (Barcelona, Girona, Central Catalonia, and Tarragona) (*χ* ^2^ = 826.39, *df* = 6, *p* < 0.001, Cramér’s *V* = 0.030). Statistically significant group differences were observed, with the group who had at least one mental diagnosis having a higher BMI (U = 6.98 x 10^8^, *p* < 0.001), number of medical comorbidities (U = 4.66 x 10^10^, *p* < 0.001), more PC visits (U = 3.93 x 10^10^, *p* < 0.001), and prescriptions of benzodiazepines (U = 4.68 x 10^8^, *p* < 0.001). They also had higher prescriptions of antipsychotics (U = 1.17 x 10^8^, *p* < 0.001) and antidepressants (U = 1.69 x 10^9^, *p* < 0.001) (Table 1).

A multivariate binary logistic regression analysis was conducted to investigate the relationship between various variables and the likelihood of having a mental health diagnosis at a PC setting during the study period. Results showed that individuals with higher numbers of medical comorbidities (OR = 1.15, 95% CI [1.14, 1.16], *p* < 0.001), female sex (OR = 1.85, 95% CI [1.78, 1.93], *p* < 0.001), and higher BMI (OR = 1.004, 95% CI [1.001, 1.007], *p* = 0.013) were associated with increased odds of having a mental health diagnosis. Smoking status was also associated with increased odds (OR = 1.09, 95% CI [1.06, 1.12], *p* < 0.001). Health care regions (OR = 1.00, 95% CI [0.99, 1.01], *p* = 0.967) and age (OR = 1.00, 95% CI [0.99, 1.01], *p* = 0.425) differences were not statistically significant. Conversely, higher socioeconomic status was associated with decreased odds of mental health diagnosis (OR = 0.94, 95% CI [0.92, 0.96], *p* < 0.001). Notably, non-Spanish nationality was associated with lower odds of having a mental health diagnosis (OR = 0.80, 95% CI [0.75, 0.86], *p* < 0.001). The model was statistically significant, with all variables except age and region showing significant associations with mental health diagnosis. The model exhibited good fit (AIC = 838548) and was statistically significant, with a reduction in deviance from 898090 (null model) to 838504 (fitted model).

## Discussion

Our results highlight the persistent and growing impact of mental health diagnoses within the observed population in PC settings during the last decades, which already preceded the COVID-19 pandemic. In line with previous studies in Europe and around the world, the most significant increases were seen in unspecific insomnia, anxiety, and depressive symptoms as well as adjustment disorders.

Insomnia, anxiety, depression, and adjustment disorders share a common underlying factor: their strong association with psychological and environmental stress [2,18,19]. Research has highlighted significant comorbidity among these conditions, with insomnia frequently co-occurring with anxiety and depression [20]. Stressful life events are known to exacerbate or trigger these conditions, supporting the notion that stress is a central factor. Adjustment disorders, in particular, are explicitly defined as maladaptive responses to identifiable psychosocial stressors, emphasizing their direct connection to stress [21]. The transdiagnostic approach to psychopathology further supports this categorization, as it identifies common underlying processes related to stress reactivity and emotion regulation across these disorders [22].

The relationship between temporal stressors and these mental health diagnoses has been thoroughly examined, with particular emphasis on the specific contextual factors of preceding economic downturns. Concomitantly, research has focused on elucidating both individual behavioural coping mechanisms and broader policy interventions that may serve to mitigate their mental health consequences in the population [23–27].

While the association of these diagnoses and the aforementioned crises have been extensively studied, the broader question of whether the observed prevalent stress-related mental health diagnoses is primarily attributable to elevated societal stress levels, enhanced public awareness, or other sociocultural factors that potentially diminish stress resilience, lies beyond the scope of this work. The complex interplay of these factors and their relative contributions to the observed trends in mental health diagnoses deserve further research.

However, it is important to consider that contemporary societal changes, including economic pressures, social media influences, and changes in family dynamics, may contribute to the rising rates of these conditions, as was highlighted in a recent report by the World Health Organization [28]. For instance, Twenge et al. (2019) highlight the role of cultural and generational shifts in increasing rates of anxiety and depression among younger populations, suggesting that broader societal changes are influencing mental health trends [29]. Accordingly, identifying protective factors, such as resilience, as a coping mechanism for stress-related mental health diagnoses could contribute to patients feeling better able to manage less chronic symptoms, providing them with more autonomy. Similarly, this may help to inform healthcare policies aimed at mental health prevention and promotion, with the overarching aim of increasing patient well-being.

In the context of these societal changes and their impact on mental health, our findings regarding immigration present an interesting counterpoint to some previous research more closely with the healthy-immigrant hypothesis [30], which suggests that recent immigrants often have better health outcomes, including mental health, compared to the native-born population. This contrasts with some previous studies suggesting increased mental health problems among immigrants. However, this finding merits a careful interpretation due to several potential confounding factors. The apparent lower prevalence of mental health diagnoses among immigrants could be influenced by underrepresentation in our sample, barriers to accessing PC, or cultural differences in help-seeking behaviours. It is also important to note that what is captured by the EHR is the nationality but not the condition of the immigrant itself. Additionally, there is the possibility that immigrants with more severe mental health problems are accessing specialized services directly, thus not forming part of our sample. Immigrant populations often face unique challenges that could impact their mental health over time, including exposure to lower socioeconomic conditions and limited healthcare access, leading to an increased risk of severe mental health disorders, as extensively reported in the literature [31,32].

Overall, our results highlight that the increasing healthcare demand for PC mental health diagnoses is being managed by a notoriously underfunded and under-resourced public PC setting. Although these conditions are generally less severe and of limited duration compared to other more severe mental illnesses, their high prevalence demands a significantly greater number of PC visits and psychotropic prescriptions compared to patients without a mental health diagnosis [33,34]. It’s crucial to interpret the observed trends in mental health diagnoses with caution. Increases in diagnoses may reflect not only changes in underlying prevalence but also shifts in awareness, reduced stigma, and evolving diagnostic practices among both patients and healthcare providers [10]. Our study primarily examines time patterns in recorded diagnoses rather than true prevalence, and this distinction is important when interpreting our results. As such, our findings suggest the potential role of psychological stress in relation to demographic and health-related factors in mental health diagnoses. Our model controlled for several variables, demonstrating the relevance of sociodemographic and clinical characteristics such as sex, and socioeconomic status in people diagnosed with a mental health condition. Importantly, other potential determinants, such as smoking habits, medical comorbidities or BMI differences, could be secondary consequences of the primary mental health diagnosis or vice versa. However, due to the limitations in the temporal availability of these data, our model could not rule out this bidirectional possibility nor establish causality.

Most of the demographic and social determinants identified in our analyses, such as female sex and lower socioeconomic status, are widely recognized as factors influencing both mental and physical health across all regions and cultures [33,35]. These social gradients in mental health underscore the necessity for a more comprehensive epidemiological approach to enhancing overall population’s mental health. Such an approach, as highlighted by Kirkbride et al. [32], should prioritize social justice and equity in access to resources that promote mental well-being. This strategy, which indirectly and in the mid to long term can optimize healthcare system resources, particularly in PC settings, aligns with the concept of treating whole populations rather than solely focusing on only high-risk individuals [32,36,37]. As previously recommended in the economic crises affecting Europe [27], this can be achieved through targeted local and global promotion and prevention strategies aimed at improving modifiable environmental factors. These strategies should focus on critical windows in the life course, particularly early life interventions, to interrupt the intergenerational transmission of mental health inequalities. Furthermore, poverty alleviation should be a central focus, given its pervasive influence on multiple social determinants of mental health. Rather than solely increasing healthcare system resources to meet rising demand, a shift towards primary prevention that addresses social determinants could yield substantial gains in population mental health. This approach requires cross-sector collaboration and investment in interventions that pay off in multiple domains, not just mental health, to create environments that promote mental well-being and prevent the onset of mental disorders [32,38].

Given the resource constraints in PC settings and the need for innovative approaches to address the rising prevalence of mental health diagnoses, it’s crucial to explore cost-efficient strategies that can augment traditional care models. One such strategy is leveraging new digital technologies to increase access to mental health care. Notably, digital mental health interventions have shown increased evidence of efficacy in monitoring and treating anxiety and depressive symptoms [39,40]. Additionally, the current surge of artificial intelligence (AI) solutions could further enhance these possibilities, particularly in optimizing population screening tools, and healthcare workflows and decision support for this increasing demand of mental health care [41]. Beyond potential interventions, our study and the mounting evidence from the COVID-19 pandemic emphasize the imperative that improving population mental health requires collaborative efforts across various societal stakeholders, extending beyond healthcare services alone [32].

There are several strengths and limitations to consider for a thorough interpretation of our results. First, it is worth noting that the study sample was derived as a control group for a larger study on patients followed in specialized mental health services, matched by age, sex, and health region. This can lead to including groups that are more highly represented among severe mental health patients. Nonetheless, our study sample closely aligns with the total Catalan population (7,629,889) in terms of sex, age, and regional distribution, as evidenced by comparison with 2019 data from the Institut d’Estadística de Catalunya (Idescat) [42]. The sex distribution in our sample (50.9% female) closely matches that of the general population (50.8%). Age distribution is comparable across all group ages; for example, middle-aged adults (45–64) represented 31.8% of our sample, compared to 26.8% of the Catalan population in 2019. Regional distribution also shows similar patterns, with slight variations due to different categorization methods. For instance, the Barcelona area comprises 66.0% of our sample compared to approximately 73.76% in the general population. These similarities strengthen the validity of our results as representative of the broader Catalan population, although some caution is warranted due to the original matching process used in sample selection.

Secondly, our dataset comprises retrospective EHRs generated continuously by various and diverse PC health professionals across Catalonia at different time points. Considering that the EHRs were sourced from a unified system, it is probable that the data entries remained consistent due to minimal changes in diagnostic and treatment protocols over time. Moreover, previous diagnoses coded under ICD-9 were seamlessly harmonized to ICD-10 standards, maintaining continuity and accuracy in the classification of diseases and health conditions. Moreover, given that most of the Catalan population has access to public health care and uses those resources, the sample size is substantial [43] and very representative of the target population.

Finally, and most importantly, despite the substantial sample size, the inherent nature of EHR-derived data presents intrinsic limitations and potential biases that are challenging to overcome, warranting consideration. The collection of sociodemographic and clinical variables at specific time points, juxtaposed with the cumulative totals of prescriptions and visit frequencies over the entire study period, may lead to temporal inconsistencies in our analyses. This discrepancy is particularly relevant for time-dependent analyses and group comparisons, despite the extensive 10-year duration of the study. A significant limitation stems from the constraints of the data available, which precluded our ability to definitively exclude the possibility of severe mental health disorders prior to 2010. Consequently, our sample may include patients with more severe diagnoses who initially presented with less severe or prodromal symptoms in earlier years, as well as similar cases in later years or those in remission. This temporal ambiguity could potentially skew our interpretation of trends over time and affect the observed associations between mental health diagnoses and other variables. Moreover, this limitation highlights the inherent challenges in capturing the full trajectory of mental health disorders, which often evolve over extended periods. Critically, our main conclusion regarding increased diagnoses across time should be interpreted with caution, as the exclusion of patients presenting to specialized services at any point during the study period may significantly impact this trend. This limitation underscores the complexity of accurately tracking mental health diagnosis patterns in primary care settings and emphasizes the need for longitudinal studies that can account for the full spectrum of care pathways.

To partially mitigate some of these issues, we prioritized using determinants less likely to fluctuate over time in our models. Despite the statistical significance of observed differences, the magnitude of these effects requires cautious interpretation, especially given the large sample size and unbalanced groups where even small differences can achieve significance. We employed a multivariate binary regression analysis to control at least some of these determinants. While our dataset includes essential sociodemographic variables such as sex, age, socioeconomic status, and sociosanitary region, other pertinent factors known to influence mental health outcomes, such as education level, ethnicity and employment status, are not routinely recorded. Future research, including all possible mental health determinants and using longitudinal designs, is necessary to better understand the causal pathways and temporal dynamics of these associations.

In conclusion, this study underscores the complex interplay of factors contributing to the growing impact of mental health diagnoses in PC settings in Catalonia, potentially reflecting broader trends across Europe and worldwide. Our findings highlight the influence of demographic and socioeconomic factors on these rates, emphasizing the need for innovative, integrated approaches that engage multiple sectors beyond healthcare. By prioritizing mental health within public health agendas and forming cross-sector partnerships, we can develop comprehensive strategies to promote mental health, prevent disorders, and improve outcomes. These findings offer a valuable template for similar initiatives globally, underscoring the importance of addressing mental health as a societal issue that extends beyond traditional healthcare boundaries.

## Data Availability

The anonymized individual-level data used in this study cannot be shared with third parties due to an agreement signed with PADRIS-AQUAS. However, aggregated data are available upon reasonable request to the corresponding author.
